# Community Readiness for Local COVID-19 Management

**DOI:** 10.3389/frma.2020.602200

**Published:** 2021-01-15

**Authors:** Riccardo Pozzo, Vania Virgili

**Affiliations:** ^1^ Department of History, Humanities and Society, University of Rome Tor Vergata, Rome, Italy; ^2^ National Institute of Nuclear Physics, Frascati, Italy

**Keywords:** community readiness, disaster risk reduction, cultural innovation, research infrastructures, responsible research and innovation, societal readiness levels, technology readiness levels

## Abstract

The experience of COVID-19 has highlighted the strategic role of local administrations, in all areas of service, in directing and coordinating actions to contain the pandemic. In this brief research report, we have interpreted the theme of the issue Open when, why, to whom? Changing challenges, perspectives, and practices in a new research culture by transferring it into a local context, namely in Italy’s inner areas, whose communities had already endured the 2016–2017 seismic swarm. We will look into the issue pragmatically, because we think that in front of a COVID-19 induced fast-changing institutional environment, science and technology studies researchers have some ideas to offer. These days, we are learning important lessons in citizen science. Today, local administrators must equip themselves with the management of infrastructures (unimaginable before COVID-19) for enforcing social distance and tracking positive cases. One of the tasks that we wish to take up is determining the levels of societal readiness and the levels of integration in society of new technologies, products, and services. The pandemic requires social and cultural innovation policies that make communities ready to respond to catastrophic events on their territory—our case-study is Italy’s inner areas—through access to data, communities of practice, co-creation, reflection, and inclusion. Finally, COVID-19 ought not to undermine the work done so far to achieve Sustainable Development Goal 1 (Poverty), 3 (Health), 4 (Education), 5 (Gender), 6 (Water), 8 (Work), 10 (Inequalities) and 16 (Peace). Pope Francis has made it clear: “This is the moment to see the poor.”

## Introduction

The experience of COVID-19 has highlighted the strategic role of local administrations, in all areas of service, in directing and coordinating actions to contain the pandemic. In this brief research report, we have interpreted the theme of the issue Open when, why, to whom? Changing challenges, perspectives, and practices in a new research culture by transferring it into a local context, namely in Italy’s inner areas, whose communities had already endured the 2016–2017 seismic swarm. We will look into the issue pragmatically because we think that in front of a COVID-19 induced fast-changing institutional environment, science and technology studies researchers have some ideas to offer to assist communities with taking highly technical decisions in crisis and relief situations.

The impact of COVID-19 on society is receiving enormous attention from whom is involved in research and innovation. The pandemic is not the first, and it will not be the last of the twenty-first century, but already today, we can consider it as the most significant science communication experience in the history of the world. In the media, we are witnessing an explosion of initiatives of citizen science, the “science of ordinary citizens” or the “science without scientists” (Irwin, 1995); and we can say that the pandemic invites us to rethink the indicators of Responsible Research and Innovation (Archibugi, 2014) for a re-determination of the effectiveness in the exchange between the knowledge of scientists and the experiential knowledge of communities (Foray, 2012). At this juncture, one of the tasks that researchers in the social sciences and humanities can take upon themselves is verifying the Societal Readiness Levels, i.e., the levels of integration into society of new technologies, products, and services (IFD-Innovation Fund Denmark, 2019).

It is up to governments to establish rules to contain the contagion, and it is up to scientists to propose recommendations based on datasets that are identified and made gradually available. Nobody can rule out that, in the future, equally contagious and more lethal viruses might endanger the lives of millions of people in every corner of the planet. For this reason, we must brace ourselves. And it is about community readiness that we want to discuss in this article.

## Materials and Methods

A community is a structure that inhabits an area with determined geomorphological, social, political, and economic characteristics (Sartori, 2017: 47). Communities are at many different stages of readiness for implementing programs, and this readiness is a significant factor in determining whether a local program can be effectively implemented and supported by the community (Edwards et al., 2000, 291).

Measures that ensure the preparedness of a community fall within the framework of health prevention and are mandatory or required by law. In contrast, the government cannot impose any of the processes that induce the readiness of a community to accept new contents and processes. In Italian inner areas, recent surveys have provided qualitative and quantitative data to establish how far communities are ready to tackle the effects of natural disasters by signing up for additional insurances, taking up new mortgages, and subscribing to further services for utilities (Russo and Scagliarini, 2017), which communities did not do in compliance with the law, but voluntarily.

We can measure the effectiveness of the exchange between the scientific community’s knowledge and the experiential knowledge of the general public according to increasingly precise indicators that range from no-awareness to professionalization—stage after stage—through denial, vague awareness, preplanning, preparation, initiation, stabilization, confirmation, and expansion (Edwards et al., 2000, 298–300). Today, the COVID-19 pandemic makes it urgent to revisit this dimension of the knowledge economy (Foray, 2006), highlighting the institutional mechanisms that make it efficient in producing cumulative and reliable knowledge as public goods.

Education, research, and innovation form a triangle that becomes a square if we add the fourth side: society. Nor can we deny the existence of injustice in the distribution of knowledge, education, and communication, what Miranda Fricker (2007) calls epistemic injustice. In this context, it is useful to keep in mind that the need for expressions of citizen science implies a connection to the “fragility of experiential knowledge,” i.e., the knowledge that—although not scientific—is produced through the experience activity of the laity. Experiential knowledge—Dominique Foray has stated—is local, since it arises from particular experiences and applies to very particular contexts. It is fragile, since not only are few people who possess it, but as it does not have a comprehensive codification, it is not easy to transmit it, and it disappears when the people who activated it disappear (Foray, 2012: 272–273).

## Discussion

Emergency management puts the usual division of roles and responsibilities under stress. Public officials must have precise knowledge of the specific normative framework in which they operate, specific mandates and associated role responsibilities, and the special normative tools contemplated by the system to deal with emergencies. It is up to local administrations to raise risk-awareness, despite the different perceptions that citizens have of its immediacy and the different conditions that make it possible to involve stakeholders. In Italy, we have found similar experiences in response to natural disasters, such as in response to the 2016–2017 seismic swarm in Emilia-Romagna (ENERGIE, 2019).

The definition of an action protocol in emergency conditions is not sufficient to guarantee the actions’ effectiveness. There is also a need for practices that mobilize the intervention of individual employees of public administrations who are coping with conditions in which chains of command and purely hierarchical-organizational relationships might be interrupted or with skills that would no longer be available in ordinary conditions. Municipalities that had already developed an emergency plan (in the wake of natural disasters) have proven to be more ready and effective in dealing with the pandemic’s specific risk conditions (Pagliacci and Russo, 2019a). In the following sections, we will discuss integrating such actions into the current understanding of community readiness and how it advances current views.

### Vulnerability

The uneven geographic distribution of COVID-19 remains an enigma in Italy, given the intense flow of movements between regions before the isolation measures. We are facing irregular patterns of geographical distribution. However, the data collected so far indicate that air pollution in the various regions (for example, the fine dust in Lombardy) determines causal links that have significant implications for the spread of the virus (Becchetti et al., 2020).

A community can be more or less resilient (Cutter et al., 2014). The resilience of a community improves by a proper assessment of local hazards and vulnerabilities. Under social and material vulnerability, “we commonly mean the exposure of some segments of the population to risk situations, understood as the uncertainty of their social and economic condition” (ISTAT-Istituto Nazionale di Statistica, 2020).

The analysis of local exposures and vulnerabilities suggests that communities tend to be spatially linked risks (UNDRR-United Nations Office for Disaster Risk Reduction, 2020). Socio-economic research can elaborate analytical insights into specific and geographically defined risks by using data with different spatial granularity produced by various official sources, to allow its use in combination with data on exposure and vulnerability (Pagliacci and Russo, 2019b).

In Italy, epidemiological data about COVID-19 are daily collected by the regional institutions that send them to the Italian Ministry of Health. The Italian Ministry of Health, in turn, sends the data to the Italian Civil Protection Department (Italian Civil Protection Department et al., 2020), which is the government agency entrusted with driving rapid response and informed decision-making during emergencies. Thanks to the accurate and quick availability of data, Italian central and local administrations are able to provide careful assessments of the severity, spread, and impact of the pandemic to implement efficient and effective response strategies, as it has been shown for many countries beyond Italy (RDA-Research Data Alliance COVID-19 Working Group, 2020).

The need for timely and accurate collection, reporting, and sharing of data within and between research communities, public health practitioners, clinicians, and policymakers has been met quite soon. The issue is building processes that can create a lasting coalition around the goals needed to reduce vulnerability. Dedicated to social and material vulnerability and resilience of communities exposed to natural hazards is Italy’s REDI consortium (an acronym for REducing Risks of Natural Disasters), which has its seat at the University of Camerino and which also includes the National Institute of Nuclear Physics, the National Institute of Geophysics and Volcanology and the Gran Sasso Science Institute. REDI is a research, innovation, and training center. Its mission is to contribute to the development of interdisciplinary research for improving preparedness and readiness to respond to disasters by communities, decreasing their recovery and recovery times. It is currently carrying out projects on re-qualified built environment, on community resilience and risk awareness, on education, training and engagement for disaster risk reduction for communities struggling to recover from natural disasters (REDI-REducing Risks of Natural Disasters, 2020).

Finally, a public debate on lessons learned from the first phases of COVID-19 management is currently taking place in Italy because the perception of a lack of coordination has emerged between political and scientific levels and institutional claim-makers, and the media (Ruiu, 2020).

### Preparedness

The reference definition for community preparedness in the face of epidemiological risks was proposed by the U.S. Centers for disease Control and Prevention in 2018 and updated in January 2019:

Community preparedness is the ability of communities to prepare for, withstand, and recover from public health incidents in both the short and long term (CDC-Centers for Disease Control and Prevention, 2019).

Administrations at national, regional, and municipal levels, as well as local and territorial stakeholders, are responsible for preparing communities to do their part in supporting the development of public health, health care, human services, mental/behavioral health, and environmental health systems that support the community preparedness. Communities need to be made aware of preventing, responding to, and recovering from incidents that adversely affect public health (CDC-Centers for Disease Control and Prevention, 2019).

### Readiness

In 2013, the International Standard Organization published the Technology Readiness Levels, a list of indicators capable of assessing the level of maturity of a given technology (ISO-International Organization for Standardization, 2019). That said, the Technology Readiness Levels (ISO-International Organization for Standardization, 2019) must be accompanied by the corresponding Societal Readiness Levels (IFD-Innovation Fund Denmark, 2019), which are a list of indicators that assess the level of social adaptation—or put in other words—that evaluate how a particular project, technology, product, process, intervention or innovation (social or technical) finds ways for integration into society.

Returning to COVID-19 and taking territory as a reference (region, metropolitan city, province, internal area), today, we know that local administrations must equip themselves with management infrastructures that were unimaginable before COVID-19 in order to comply with social distancing precautions and be effective with positive case tracking.

## Results

Community readiness is about fostering epistemic responsibility, whose effectiveness can be measured in terms of community engagement and accountability relationships. At the local level, the availability of correct information to people with relevant competencies and skills, at the right time and in the right form, is a key dimension in coping with emergencies. Typically, conflicts arise about whether, how, and when to distribute information. In this respect, Italian inner areas have faced critical situations. It has been shown that a proper assessment of local hazards and vulnerabilities can enhance community resilience (Pagliacci and Russo, 2019a).

At the European level, Pan-European Privacy-Preserving Proximity Tracing (PEPP-PT) and Decentralized Privacy-Preserving Proximity Tracing (DP3T) have become an issue. Both the European Parliament and the European Commission have adopted a firm position on safeguarding privacy in the fight against COVID-19.

According to an SWG survey published by Corriere della Sera at the climax of the COVID-19 spread in Italy, on March 30, 2020, it appears that 1) 63% of Italians agree that the state can control the movements of citizens even without their consent; 2) 64% agree on the hypothesis of putting the electronic bracelet on people who are in quarantine; 3) 67% accepted that mobile phones are used to check whether or not people are complying with the bans; and finally 4) that 74% have nothing to object to the use of drones to control the movement of people on the street (Arachi, 2020).

### Legal Basis

The reference text is paragraphs 25–26 of the Siracusa Principles on the Limitation and Derogation of Provisions in the International Covenant on Civil and Political Rights:

[§25] Public health may be invoked as a ground for limiting certain rights in order to allow a state to take measures dealing with a serious threat to the health of the population or individual members of the population. These measures must be specifically aimed at preventing disease or injury or providing care for the sick and injured. [§26] Due regard shall be had to the international health regulations of the World Health Organization (Siracusa, 1985).

Communities should consider biometric surveillance as a temporary measure taken during a state of emergency, to be repealed once the emergency is over. Nevertheless, temporary measures have “the bad habit of becoming lasting, especially since there is always a new emergency on the horizon” (Harari, 2020).

### Social Innovation

As the emergency increases, the need for transparency grows. If society’s readiness for a specific social or technical solution is low, measures should induce a natural transition toward social adaptation. The lower the social adaptation, the better the transition plan must be. SRL 1 is the lowest, and SRL 9 is the highest level:

SRL 1—identifying problem and identifying societal readiness SRL 2—formulation of problem, proposed solution(s) and potential impact, expected societal readiness; identifying relevant stakeholders for the project SRL 3—initial testing of proposed solution(s) together with relevant stakeholders SRL 4—problem validated through pilot testing in relevant environment to substantiate proposed impact and societal readiness SRL 5—proposed solution(s) validated, now by relevant stakeholders in the area SRL 6—solution(s) demonstrated in relevant environment and in co-operation with relevant stakeholders to gain initial feedback on potential impact SRL 7—refinement of project and/or solution and, if needed, retesting in relevant environment with relevant stakeholders SRL 8—proposed solution(s) as well as a plan for societal adaptation complete and qualified SRL 9—actual project solution(s) proven in relevant environment (IFD-Innovation Fund Denmark, 2019).

In the case of natural disasters, and such is the pandemic, at issue is how to set into motion social and cultural innovation processes that prepare communities to respond to catastrophic events on their territory through access to data, participation in communities of practice, co-creation, reflection, and inclusion (Pozzo et al., 2020).

### Cultural Innovation

Culture is tradition—people say after the Analects of Confucius (7.1)—and does not need innovation. Today we know critical cultural innovation processes, which are recharged and reinvigorated through social innovation experiences and technological innovation paths. In order to identify useful indicators for measuring cultural innovation, an interesting approach is the one that takes up the idea of the “joint creation of value by the producer and the consumer, allowing the consumer to contribute to the construction of the service experience to adapt it to their needs” (Prahalad and Venkatram, 2000, 83).

Cultural innovation looks at reflexivity, at the individual’s ability to distinguish some aspects in the indiscriminate mass of the flow of experiential content, isolate them, and focus on them (Archer, 2003). Cultural innovation also looks at inclusion within the diverse communities of civil societies due to shared experiences, common goods, and spaces for exchange (Pozzo and Virgili, 2016).

Today, more than ever, the importance of culture and creativity for society is evident. The availability of cultural content contributes to the acceptance of the other, dialogue, sharing, health, and mental well-being. It is clear to everyone that the crisis caused by COVID-19 is particularly dramatic for the cultural and creative sector, due to the sudden collapse of use and the consequent massive loss of revenue opportunities, especially for the most fragile actors. The COVID-19 crisis creates a structural threat to many companies and workers’ survival, dedicated to cultural and creative production. “Sustainable business models during and after the initial crisis are imperative for the sector’s survival. Leaving behind the more fragile part of the sector could cause irreparable economic and social damage” (OECD-Organisation for Economic Co-Operation and Development, 2020).

## Conclusion

The pandemic is persisting, and the world is about to enter into the second year of struggle. The winter 2020/21 needs a great effort of responsibility and participation. For this reason, the pandemic invites us to urgently rethink the paradigm of the six keys indicated by the European Commission for Responsible Research and Innovation, which are: “engagement of citizens, gender equality, formal and non-formal science education, open science, research ethics and research integrity, governance framework” (Archibugi, 2014). Working on participatory approaches fueled by social and cultural innovation processes related to accessing data, creating communities of practices, establishing the boundaries of group use (Floridi, 2014), while fostering individual processes of reflection and collective processes of inclusion (Pozzo et al., 2020) can boost community readiness for local COVID-19 management.

It is necessary to reflect so that the pandemic’s emergency does not undermine the work done so far to achieve in 2030 the seventeen United Nations Sustainable Development Goals. Sustainable development satisfies the needs of the present generation without compromising the satisfaction of those of future generations. Among the most probable effects of COVID-19, we might look at increases in poverty of the vulnerable population due to loss of income, closure of micro, small and medium-sized enterprises, increases in unemployment and impoverishment, and difficulties in accessing quality education, which will be most consequential for women whose emancipation will be slowed down (Braun et al., 2020). Hence, we are concerned about goals 1 (end poverty), 3 (health and well-being), 4 (quality education), 5 (gender equality), 6 (water and hygiene), 8 (growth and employment), 10 (reducing inequalities), and 16 (peace and justice) (UNSDSN-United Nations, Sustainable Development Solutions Network, 2020).

The United Nations is calling for global agreement to tackle the pandemic crisis, which “risks erasing decades of progress in the fight against poverty and exacerbating the already high levels of inequality in and between countries” (UNSDSN-United Nations, Sustainable Development Solutions Network, 2020). Local administrations are the first to work on community readiness and reduce inequalities, which is also the exhortation of Pope Francis:

The coronavirus disease 2019 pandemic has illuminated inequities that have put poor people—in both low-income nations and in rich countries—at the greatest risk of suffering. Pope Francis recently pointed to that in an interview: “This is the moment to see the poor” (Braun et al., 2020, 214).

## Data Availability

The original contributions presented in this brief research report are reflections on the works listed among the references. Further inquiries can be directed to the corresponding author.
